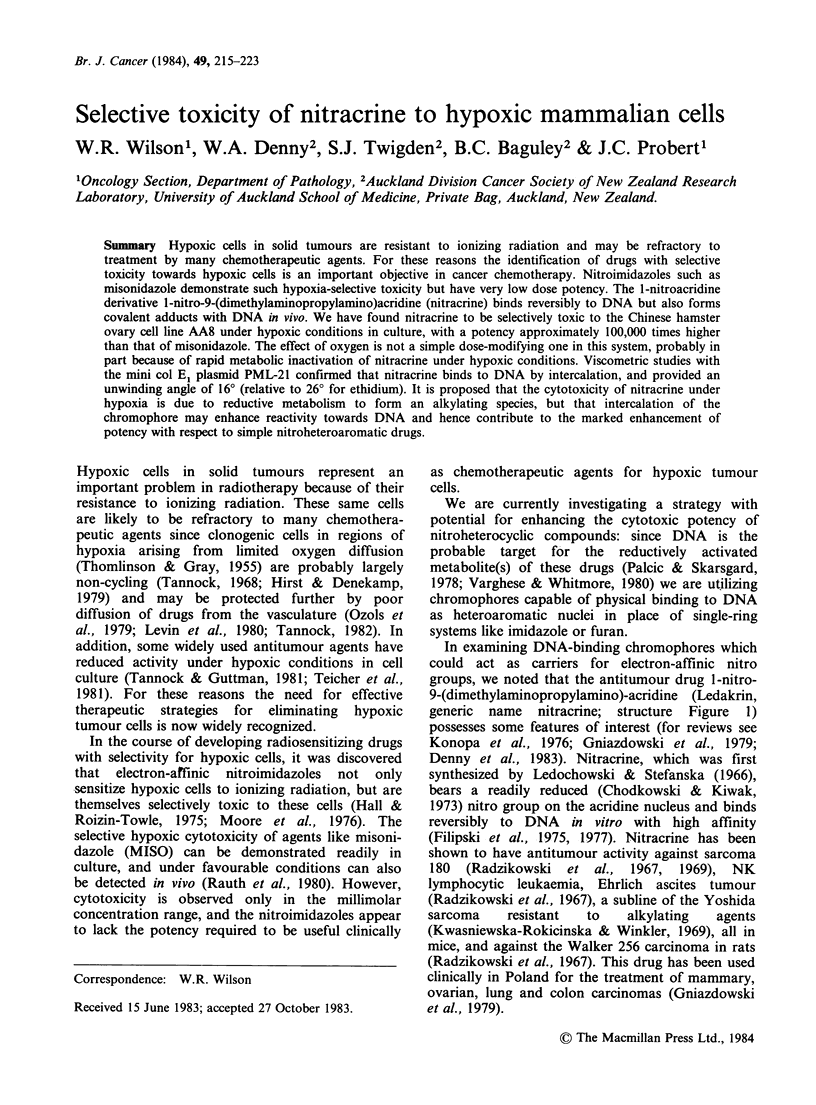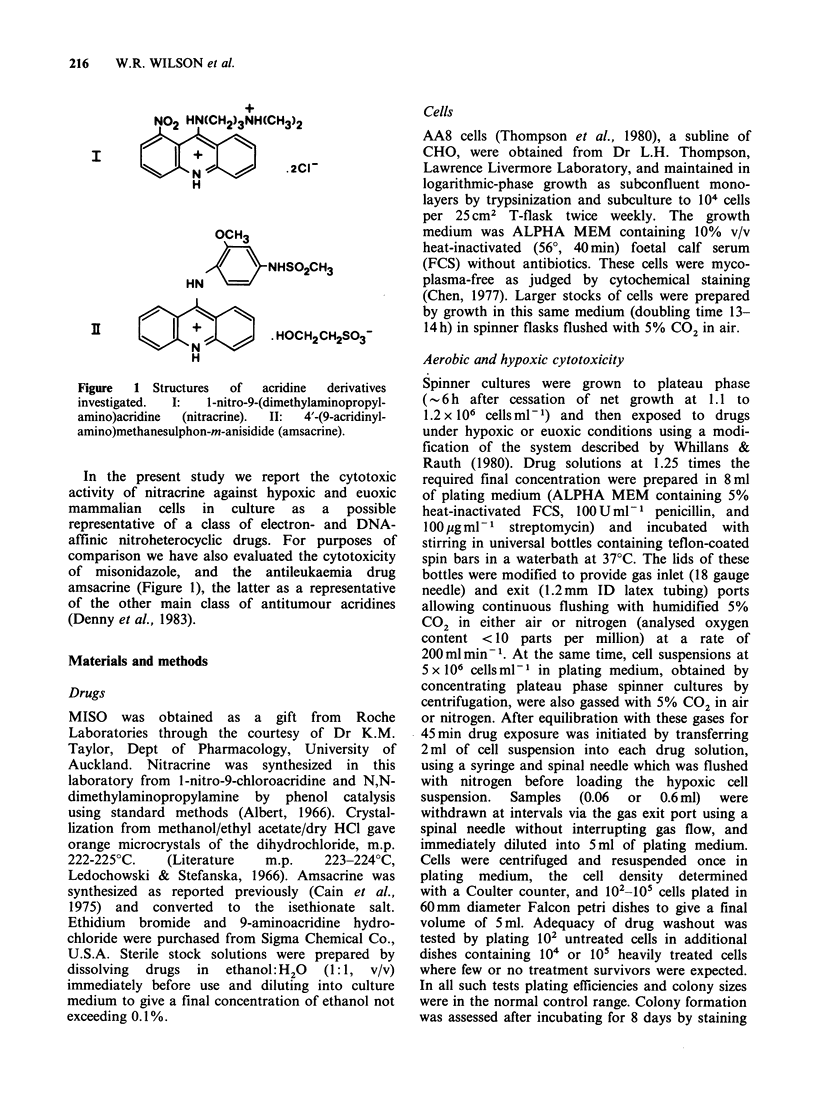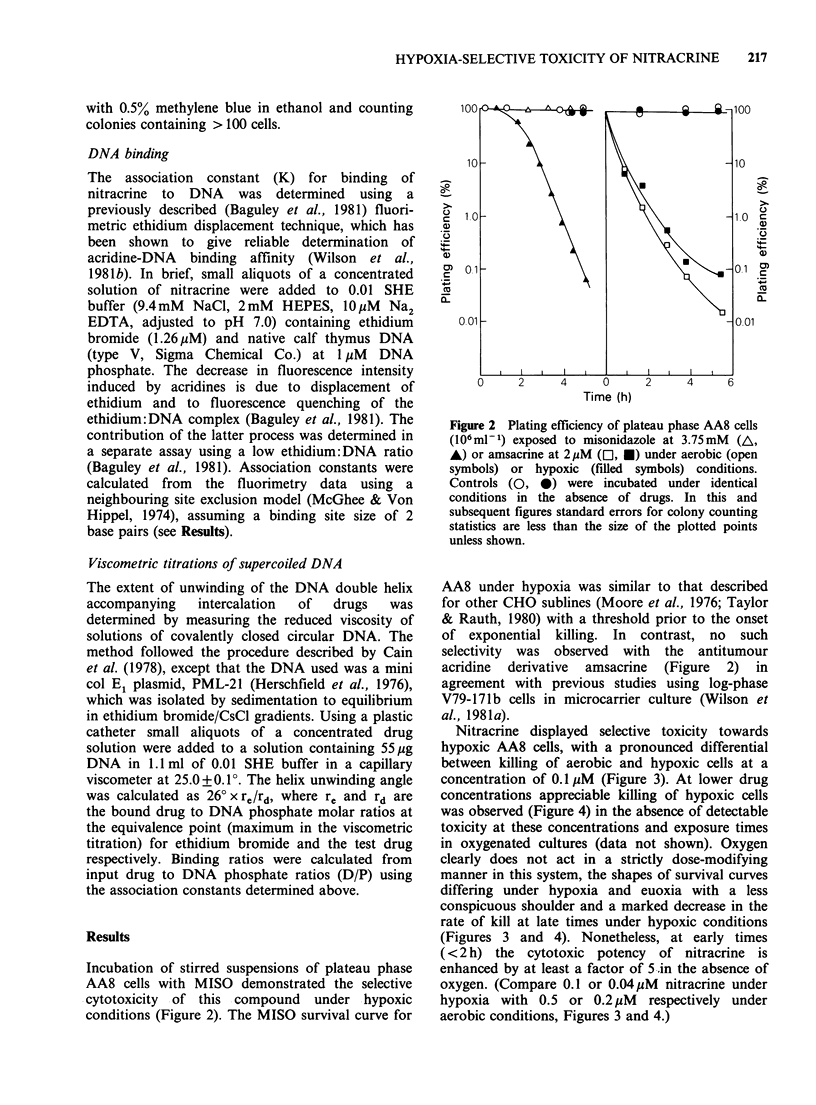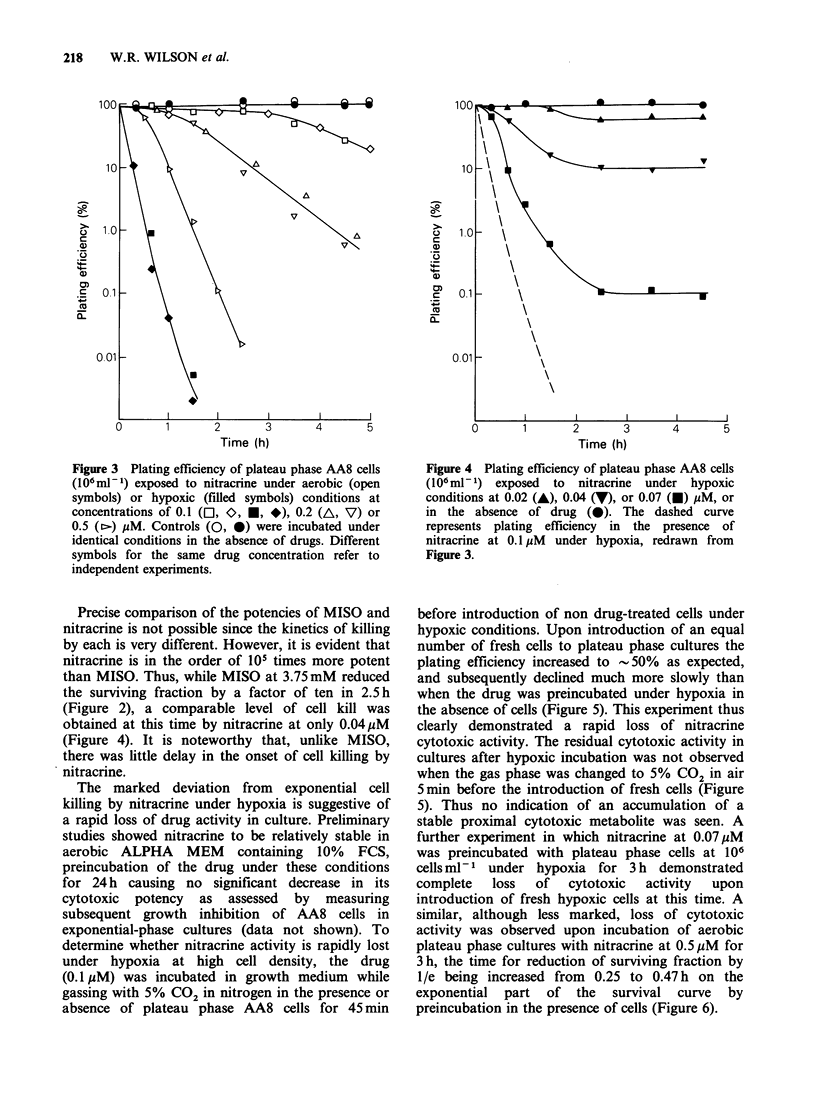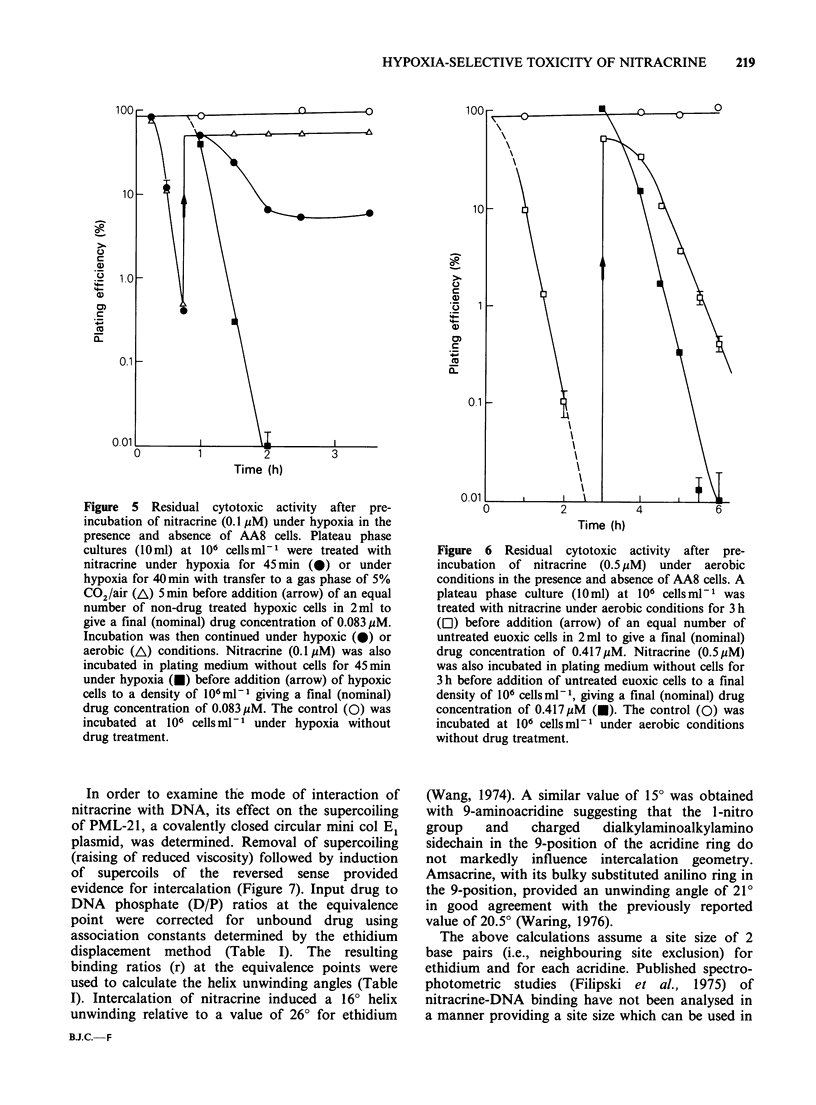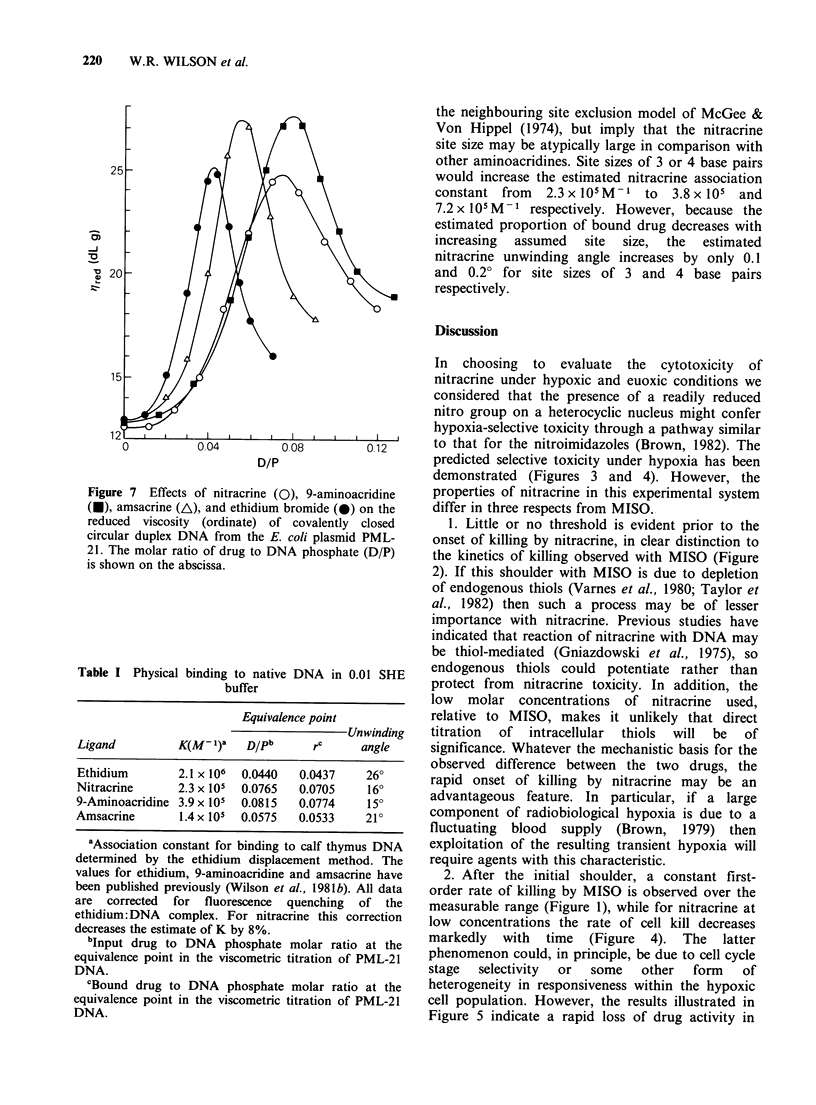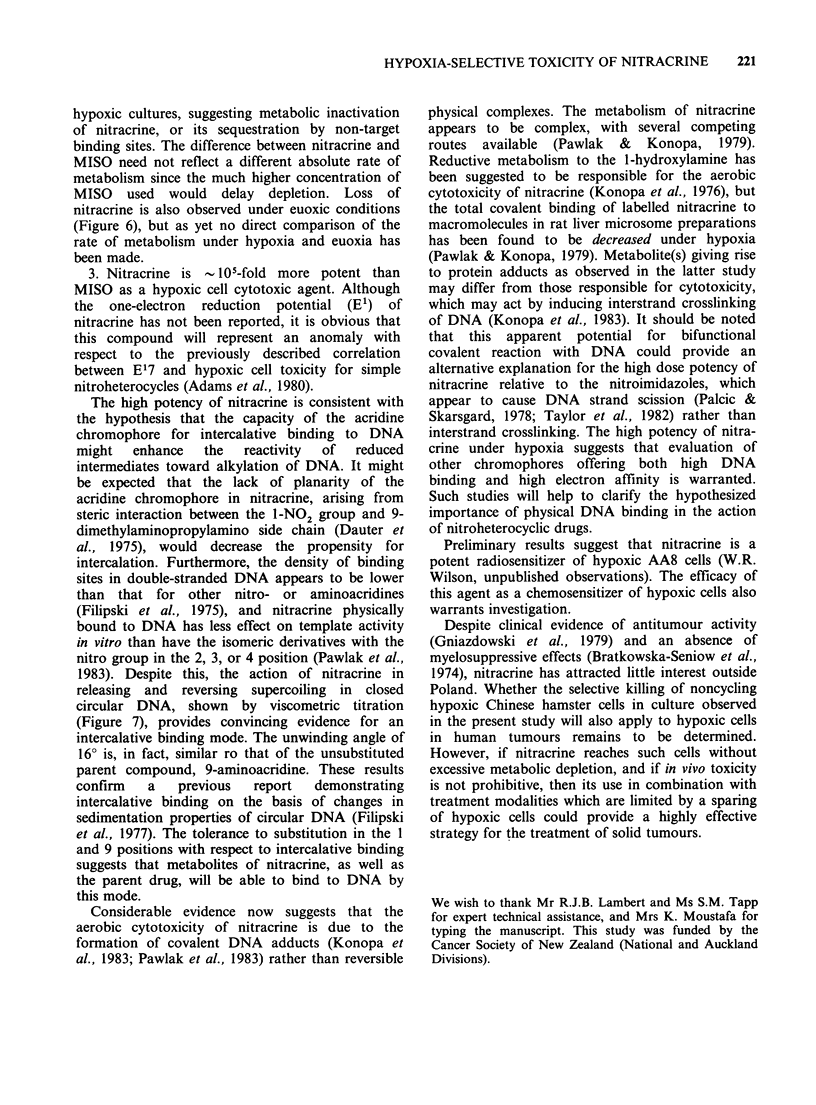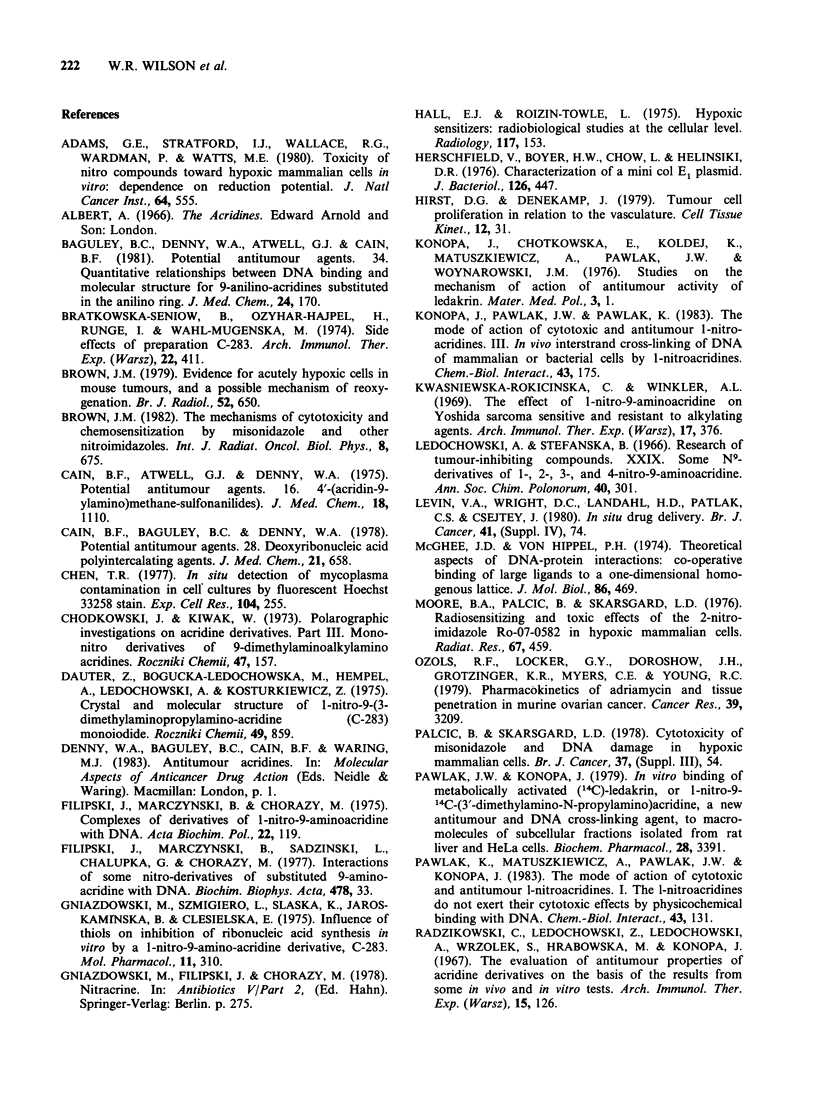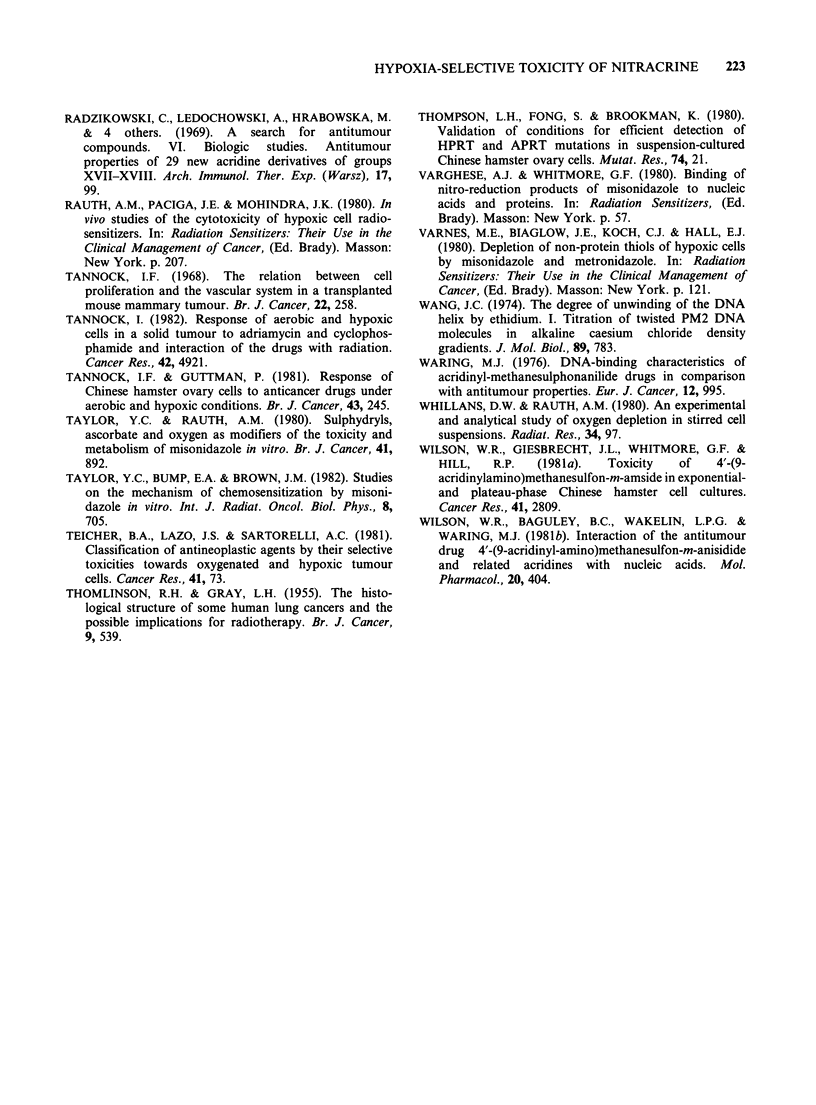# Selective toxicity of nitracrine to hypoxic mammalian cells.

**DOI:** 10.1038/bjc.1984.34

**Published:** 1984-02

**Authors:** W. R. Wilson, W. A. Denny, S. J. Twigden, B. C. Baguley, J. C. Probert

## Abstract

Hypoxic cells in solid tumours are resistant to ionizing radiation and may be refractory to treatment by many chemotherapeutic agents. For these reasons the identification of drugs with selective toxicity towards hypoxic cells is an important objective in cancer chemotherapy. Nitroimidazoles such as misonidazole demonstrate such hypoxia-selective toxicity but have very low dose potency. The 1-nitroacridine derivative 1-nitro-9-(dimethylaminopropylamino)acridine (nitracrine) binds reversibly to DNA but also forms covalent adducts with DNA in vivo. We have found nitracrine to be selectively toxic to the Chinese hamster ovary cell line AA8 under hypoxic conditions in culture, with a potency approximately 100,000 times higher than that of misonidazole. The effect of oxygen is not a simple dose-modifying one in this system, probably in part because of rapid metabolic inactivation of nitracrine under hypoxic conditions. Viscometric studies with the mini col E1 plasmid PML-21 confirmed that nitracrine binds to DNA by intercalation, and provided an unwinding angle of 16 degrees (relative to 26 degrees for ethidium). It is proposed that the cytotoxicity of nitracrine under hypoxia is due to reductive metabolism to form an alkylating species, but that intercalation of the chromophore may enhance reactivity towards DNA and hence contribute to the marked enhancement of potency with respect to simple nitroheteroaromatic drugs.


*Br. J. Cancer* (1984), **49**, 215–223

### Selective toxicity of nitracrine to hypoxic mammalian cells

W.R. Wilson[1], W.A. Denny[2], S.J. Twigden[2], B.C. Baguley[2] & J.C. Probert[1]

[1]*Oncology Section, Department of Pathology,* [2]*Auckland Division Cancer Society of New Zealand Research Laboratory, University of Auckland School of Medicine, Private Bag, Auckland, New Zealand.*

**Summary** Hypoxic cells in solid tumours are resistant to ionizing radiation and may be refractory to treatment by many chemotherapeutic agents. For these reasons the identification of drugs with selective toxicity towards hypoxic cells is an important objective in cancer chemotherapy. Nitroimidazoles such as misonidazole demonstrate such hypoxia-selective toxicity but have very low dose potency. The 1-nitroacridine derivative 1-nitro-9-(dimethylaminopropylamino)acridine (nitracrine) binds reversibly to DNA but also forms covalent adducts with DNA *in vivo*. We have found nitracrine to be selectively toxic to the Chinese hamster ovary cell line AA8 under hypoxic conditions in culture, with a potency approximately 100,000 times higher than that of misonidazole. The effect of oxygen is not a simple dose-modifying one in this system, probably in part because of rapid metabolic inactivation of nitracrine under hypoxic conditions. Viscometric studies with the mini col $E_1$ plasmid PML-21 confirmed that nitracrine binds to DNA by intercalation, and provided an unwinding angle of 16° (relative to 26° for ethidium). It is proposed that the cytotoxicity of nitracrine under hypoxia is due to reductive metabolism to form an alkylating species, but that intercalation of the chromophore may enhance reactivity towards DNA and hence contribute to the marked enhancement of potency with respect to simple nitroheteroaromatic drugs.

Hypoxic cells in solid tumours represent an important problem in radiotherapy because of their resistance to ionizing radiation. These same cells are likely to be refractory to many chemotherapeutic agents since clonogenic cells in regions of hypoxia arising from limited oxygen diffusion (Thomlinson & Gray, 1955) are probably largely non-cycling (Tannock, 1968; Hirst & Denekamp, 1979) and may be protected further by poor diffusion of drugs from the vasculature (Ozols *et al.*, 1979; Levin *et al.*, 1980; Tannock, 1982). In addition, some widely used antitumour agents have reduced activity under hypoxic conditions in cell culture (Tannock & Guttman, 1981; Teicher *et al.*, 1981). For these reasons the need for effective therapeutic strategies for eliminating hypoxic tumour cells is now widely recognized.

In the course of developing radiosensitizing drugs with selectivity for hypoxic cells, it was discovered that electron-affinic nitroimidazoles not only sensitize hypoxic cells to ionizing radiation, but are themselves selectively toxic to these cells (Hall & Roizin-Towle, 1975; Moore *et al.*, 1976). The selective hypoxic cytotoxicity of agents like misonidazole (MISO) can be demonstrated readily in culture, and under favourable conditions can also be detected *in vivo* (Rauth *et al.*, 1980). However, cytotoxicity is observed only in the millimolar concentration range, and the nitroimidazoles appear to lack the potency required to be useful clinically as chemotherapeutic agents for hypoxic tumour cells.

We are currently investigating a strategy with potential for enhancing the cytotoxic potency of nitroheterocyclic compounds: since DNA is the probable target for the reductively activated metabolite(s) of these drugs (Palcic & Skarsgard, 1978; Varghese & Whitmore, 1980) we are utilizing chromophores capable of physical binding to DNA as heteroaromatic nuclei in place of single-ring systems like imidazole or furan.

In examining DNA-binding chromophores which could act as carriers for electron-affinic nitro groups, we noted that the antitumour drug 1-nitro-9-(dimethylaminopropylamino)-acridine (Ledakrin, generic name nitracrine; structure Figure 1) possesses some features of interest (for reviews see Konopa *et al.*, 1976; Gniazdowski *et al.*, 1979; Denny *et al.*, 1983). Nitracrine, which was first synthesized by Ledochowski & Stefanska (1966), bears a readily reduced (Chodkowski & Kiwak, 1973) nitro group on the acridine nucleus and binds reversibly to DNA *in vitro* with high affinity (Filipski *et al.*, 1975, 1977). Nitracrine has been shown to have antitumour activity against sarcoma 180 (Radzikowski *et al.*, 1967, 1969), NK lymphocytic leukaemia, Ehrlich ascites tumour (Radzikowski *et al.*, 1967), a subline of the Yoshida sarcoma resistant to alkylating agents (Kwasniewska-Rokicinska & Winkler, 1969), all in mice, and against the Walker 256 carcinoma in rats (Radzikowski *et al.*, 1967). This drug has been used clinically in Poland for the treatment of mammary, ovarian, lung and colon carcinomas (Gniazdowski *et al.*, 1979).

Correspondence: W.R. Wilson

Received 15 June 1983; accepted 27 October 1983.

© The Macmillan Press Ltd., 1984

**Figure 1** Structures of acridine derivatives investigated. I: 1-nitro-9-(dimethylaminopropylamino)acridine (nitracrine). II: 4′-(9-acridinylamino)methanesulphon-*m*-anisidide (amsacrine).

In the present study we report the cytotoxic activity of nitracrine against hypoxic and euoxic mammalian cells in culture as a possible representative of a class of electron- and DNA-affinic nitroheterocyclic drugs. For purposes of comparison we have also evaluated the cytotoxicity of misonidazole, and the antileukaemia drug amsacrine (Figure 1), the latter as a representative of the other main class of antitumour acridines (Denny *et al.*, 1983).

### Materials and methods

### *Drugs*

MISO was obtained as a gift from Roche Laboratories through the courtesy of Dr K.M. Taylor, Dept of Pharmacology, University of Auckland. Nitracrine was synthesized in this laboratory from 1-nitro-9-chloroacridine and N,N-dimethylaminopropylamine by phenol catalysis using standard methods (Albert, 1966). Crystallization from methanol/ethyl acetate/dry HCl gave orange microcrystals of the dihydrochloride, m.p. 222-225°C. (Literature m.p. 223–224°C, Ledochowski & Stefanska, 1966). Amsacrine was synthesized as reported previously (Cain *et al.*, 1975) and converted to the isethionate salt. Ethidium bromide and 9-aminoacridine hydrochloride were purchased from Sigma Chemical Co., U.S.A. Sterile stock solutions were prepared by dissolving drugs in ethanol:$H_2O$ (1:1, v/v) immediately before use and diluting into culture medium to give a final concentration of ethanol not exceeding 0.1%.

### *Cells*

AA8 cells (Thompson *et al.*, 1980), a subline of CHO, were obtained from Dr L.H. Thompson, Lawrence Livermore Laboratory, and maintained in logarithmic-phase growth as subconfluent monolayers by trypsinization and subculture to $10^4$ cells per $25\,cm^2$ T-flask twice weekly. The growth medium was ALPHA MEM containing 10% v/v heat-inactivated (56°, 40 min) foetal calf serum (FCS) without antibiotics. These cells were mycoplasma-free as judged by cytochemical staining (Chen, 1977). Larger stocks of cells were prepared by growth in this same medium (doubling time 13–14 h) in spinner flasks flushed with 5% $CO_2$ in air.

### *Aerobic and hypoxic cytotoxicity*

Spinner cultures were grown to plateau phase (~6 h after cessation of net growth at 1.1 to $1.2\times10^6$ cells $ml^{-1}$) and then exposed to drugs under hypoxic or euoxic conditions using a modification of the system described by Whillans & Rauth (1980). Drug solutions at 1.25 times the required final concentration were prepared in 8 ml of plating medium (ALPHA MEM containing 5% heat-inactivated FCS, 100 U $ml^{-1}$ penicillin, and 100 $\mu g\,ml^{-1}$ streptomycin) and incubated with stirring in universal bottles containing teflon-coated spin bars in a waterbath at 37°C. The lids of these bottles were modified to provide gas inlet (18 gauge needle) and exit (1.2 mm ID latex tubing) ports allowing continuous flushing with humidified 5% $CO_2$ in either air or nitrogen (analysed oxygen content $<10$ parts per million) at a rate of 200 ml $min^{-1}$. At the same time, cell suspensions at $5\times10^6$ cells $ml^{-1}$ in plating medium, obtained by concentrating plateau phase spinner cultures by centrifugation, were also gassed with 5% $CO_2$ in air or nitrogen. After equilibration with these gases for 45 min drug exposure was initiated by transferring 2 ml of cell suspension into each drug solution, using a syringe and spinal needle which was flushed with nitrogen before loading the hypoxic cell suspension. Samples (0.06 or 0.6 ml) were withdrawn at intervals via the gas exit port using a spinal needle without interrupting gas flow, and immediately diluted into 5 ml of plating medium. Cells were centrifuged and resuspended once in plating medium, the cell density determined with a Coulter counter, and $10^2$–$10^5$ cells plated in 60 mm diameter Falcon petri dishes to give a final volume of 5 ml. Adequacy of drug washout was tested by plating $10^2$ untreated cells in additional dishes containing $10^4$ or $10^5$ heavily treated cells where few or no treatment survivors were expected. In all such tests plating efficiencies and colony sizes were in the normal control range. Colony formation was assessed after incubating for 8 days by staining with 0.5% methylene blue in ethanol and counting colonies containing >100 cells.

*DNA binding*

The association constant (K) for binding of nitracrine to DNA was determined using a previously described (Baguley *et al.*, 1981) fluorimetric ethidium displacement technique, which has been shown to give reliable determination of acridine-DNA binding affinity (Wilson *et al.*, 1981*b*). In brief, small aliquots of a concentrated solution of nitracrine were added to 0.01 SHE buffer (9.4 mM NaCl, 2 mM HEPES, 10 μM $Na_2$ EDTA, adjusted to pH 7.0) containing ethidium bromide (1.26 μM) and native calf thymus DNA (type V, Sigma Chemical Co.) at 1 μM DNA phosphate. The decrease in fluorescence intensity induced by acridines is due to displacement of ethidium and to fluorescence quenching of the ethidium:DNA complex (Baguley *et al.*, 1981). The contribution of the latter process was determined in a separate assay using a low ethidium:DNA ratio (Baguley *et al.*, 1981). Association constants were calculated from the fluorimetry data using a neighbouring site exclusion model (McGhee & Von Hippel, 1974), assuming a binding site size of 2 base pairs (see **Results**).

*Viscometric titrations of supercoiled DNA*

The extent of unwinding of the DNA double helix accompanying intercalation of drugs was determined by measuring the reduced viscosity of solutions of covalently closed circular DNA. The method followed the procedure described by Cain *et al.* (1978), except that the DNA used was a mini col $E_1$ plasmid, PML-21 (Herschfield *et al.*, 1976), which was isolated by sedimentation to equilibrium in ethidium bromide/CsCl gradients. Using a plastic catheter small aliquots of a concentrated drug solution were added to a solution containing 55 μg DNA in 1.1 ml of 0.01 SHE buffer in a capillary viscometer at 25.0±0.1°. The helix unwinding angle was calculated as $26° \times r_e/r_d$, where $r_e$ and $r_d$ are the bound drug to DNA phosphate molar ratios at the equivalence point (maximum in the viscometric titration) for ethidium bromide and the test drug respectively. Binding ratios were calculated from input drug to DNA phosphate ratios (D/P) using the association constants determined above.

### Results

Incubation of stirred suspensions of plateau phase AA8 cells with MISO demonstrated the selective cytotoxicity of this compound under hypoxic conditions (Figure 2). The MISO survival curve for

**Figure 2** Plating efficiency of plateau phase AA8 cells ($10^6$ ml$^{-1}$) exposed to misonidazole at 3.75 mM (△, ▲) or amsacrine at 2 μM (□, ■) under aerobic (open symbols) or hypoxic (filled symbols) conditions. Controls (○, ●) were incubated under identical conditions in the absence of drugs. In this and subsequent figures standard errors for colony counting statistics are less than the size of the plotted points unless shown.

AA8 under hypoxia was similar to that described for other CHO sublines (Moore *et al.*, 1976; Taylor & Rauth, 1980) with a threshold prior to the onset of exponential killing. In contrast, no such selectivity was observed with the antitumour acridine derivative amsacrine (Figure 2) in agreement with previous studies using log-phase V79-171b cells in microcarrier culture (Wilson *et al.*, 1981*a*).

Nitracrine displayed selective toxicity towards hypoxic AA8 cells, with a pronounced differential between killing of aerobic and hypoxic cells at a concentration of 0.1 μM (Figure 3). At lower drug concentrations appreciable killing of hypoxic cells was observed (Figure 4) in the absence of detectable toxicity at these concentrations and exposure times in oxygenated cultures (data not shown). Oxygen clearly does not act in a strictly dose-modifying manner in this system, the shapes of survival curves differing under hypoxia and euoxia with a less conspicuous shoulder and a marked decrease in the rate of kill at late times under hypoxic conditions (Figures 3 and 4). Nonetheless, at early times (<2 h) the cytotoxic potency of nitracrine is enhanced by at least a factor of 5 in the absence of oxygen. (Compare 0.1 or 0.04 μM nitracrine under hypoxia with 0.5 or 0.2 μM respectively under aerobic conditions, Figures 3 and 4.)

**Figure 3** Plating efficiency of plateau phase AA8 cells ($10^6 ml^{-1}$) exposed to nitracrine under aerobic (open symbols) or hypoxic (filled symbols) conditions at concentrations of 0.1 (□, ◇, ■, ◆), 0.2 (△, ▽) or 0.5 (▷) μM. Controls (○, ●) were incubated under identical conditions in the absence of drugs. Different symbols for the same drug concentration refer to independent experiments.

**Figure 4** Plating efficiency of plateau phase AA8 cells ($10^6 ml^{-1}$) exposed to nitracrine under hypoxic conditions at 0.02 (▲), 0.04 (▼), or 0.07 (■) μM, or in the absence of drug (●). The dashed curve represents plating efficiency in the presence of nitracrine at 0.1 μM under hypoxia, redrawn from Figure 3.

Precise comparison of the potencies of MISO and nitracrine is not possible since the kinetics of killing by each is very different. However, it is evident that nitracrine is in the order of $10^5$ times more potent than MISO. Thus, while MISO at 3.75 mM reduced the surviving fraction by a factor of ten in 2.5 h (Figure 2), a comparable level of cell kill was obtained at this time by nitracrine at only 0.04 μM (Figure 4). It is noteworthy that, unlike MISO, there was little delay in the onset of cell killing by nitracrine.

The marked deviation from exponential cell killing by nitracrine under hypoxia is suggestive of a rapid loss of drug activity in culture. Preliminary studies showed nitracrine to be relatively stable in aerobic ALPHA MEM containing 10% FCS, preincubation of the drug under these conditions for 24 h causing no significant decrease in its cytotoxic potency as assessed by measuring subsequent growth inhibition of AA8 cells in exponential-phase cultures (data not shown). To determine whether nitracrine activity is rapidly lost under hypoxia at high cell density, the drug (0.1 μM) was incubated in growth medium while gassing with 5% $CO_2$ in nitrogen in the presence or absence of plateau phase AA8 cells for 45 min before introduction of non drug-treated cells under hypoxic conditions. Upon introduction of an equal number of fresh cells to plateau phase cultures the plating efficiency increased to ~50% as expected, and subsequently declined much more slowly than when the drug was preincubated under hypoxia in the absence of cells (Figure 5). This experiment thus clearly demonstrated a rapid loss of nitracrine cytotoxic activity. The residual cytotoxic activity in cultures after hypoxic incubation was not observed when the gas phase was changed to 5% $CO_2$ in air 5 min before the introduction of fresh cells (Figure 5). Thus no indication of an accumulation of a stable proximal cytotoxic metabolite was seen. A further experiment in which nitracrine at 0.07 μM was preincubated with plateau phase cells at $10^6$ cells $ml^{-1}$ under hypoxia for 3 h demonstrated complete loss of cytotoxic activity upon introduction of fresh hypoxic cells at this time. A similar, although less marked, loss of cytotoxic activity was observed upon incubation of aerobic plateau phase cultures with nitracrine at 0.5 μM for 3 h, the time for reduction of surviving fraction by 1/e being increased from 0.25 to 0.47 h on the exponential part of the survival curve by preincubation in the presence of cells (Figure 6).

**Figure 5** Residual cytotoxic activity after pre-incubation of nitracrine (0.1 $\mu$M) under hypoxia in the presence and absence of AA8 cells. Plateau phase cultures (10 ml) at $10^6$ cells $ml^{-1}$ were treated with nitracrine under hypoxia for 45 min (●) or under hypoxia for 40 min with transfer to a gas phase of 5% $CO_2$/air (△) 5 min before addition (arrow) of an equal number of non-drug treated hypoxic cells in 2 ml to give a final (nominal) drug concentration of 0.083 $\mu$M. Incubation was then continued under hypoxic (●) or aerobic (△) conditions. Nitracrine (0.1 $\mu$M) was also incubated in plating medium without cells for 45 min under hypoxia (■) before addition (arrow) of hypoxic cells to a density of $10^6 ml^{-1}$ giving a final (nominal) drug concentration of 0.083 $\mu$M. The control (○) was incubated at $10^6$ cells $ml^{-1}$ under hypoxia without drug treatment.

**Figure 6** Residual cytotoxic activity after pre-incubation of nitracrine (0.5 $\mu$M) under aerobic conditions in the presence and absence of AA8 cells. A plateau phase culture (10 ml) at $10^6$ cells $ml^{-1}$ was treated with nitracrine under aerobic conditions for 3 h (□) before addition (arrow) of an equal number of untreated euoxic cells in 2 ml to give a final (nominal) drug concentration of 0.417 $\mu$M. Nitracrine (0.5 $\mu$M) was also incubated in plating medium without cells for 3 h before addition of untreated euoxic cells to a final density of $10^6$ cells $ml^{-1}$, giving a final (nominal) drug concentration of 0.417 $\mu$M (■). The control (○) was incubated at $10^6$ cells $ml^{-1}$ under aerobic conditions without drug treatment.

In order to examine the mode of interaction of nitracrine with DNA, its effect on the supercoiling of PML-21, a covalently closed circular mini col $E_1$ plasmid, was determined. Removal of supercoiling (raising of reduced viscosity) followed by induction of supercoils of the reversed sense provided evidence for intercalation (Figure 7). Input drug to DNA phosphate (D/P) ratios at the equivalence point were corrected for unbound drug using association constants determined by the ethidium displacement method (Table I). The resulting binding ratios (r) at the equivalence points were used to calculate the helix unwinding angles (Table I). Intercalation of nitracrine induced a 16° helix unwinding relative to a value of 26° for ethidium (Wang, 1974). A similar value of 15° was obtained with 9-aminoacridine suggesting that the 1-nitro group and charged dialkylaminoalkylamino sidechain in the 9-position of the acridine ring do not markedly influence intercalation geometry. Amsacrine, with its bulky substituted anilino ring in the 9-position, provided an unwinding angle of 21° in good agreement with the previously reported value of 20.5° (Waring, 1976).

The above calculations assume a site size of 2 base pairs (i.e., neighbouring site exclusion) for ethidium and for each acridine. Published spectrophotometric studies (Filipski *et al.*, 1975) of nitracrine-DNA binding have not been analysed in a manner providing a site size which can be used in

**Figure 7** Effects of nitracrine (○), 9-aminoacridine (■), amsacrine (△), and ethidium bromide (●) on the reduced viscosity (ordinate) of covalently closed circular duplex DNA from the *E. coli* plasmid PML-21. The molar ratio of drug to DNA phosphate (D/P) is shown on the abscissa.

**Table I** Physical binding to native DNA in 0.01 SHE buffer

| *Ligand* | *K(M⁻¹)*[a] | *Equivalence point D/P*[b] | *Equivalence point r*[c] | *Unwinding angle* |
|---|---|---|---|---|
| Ethidium | $2.1 \times 10^6$ | 0.0440 | 0.0437 | 26° |
| Nitracrine | $2.3 \times 10^5$ | 0.0765 | 0.0705 | 16° |
| 9-Aminoacridine | $3.9 \times 10^5$ | 0.0815 | 0.0774 | 15° |
| Amsacrine | $1.4 \times 10^5$ | 0.0575 | 0.0533 | 21° |

[a]Association constant for binding to calf thymus DNA determined by the ethidium displacement method. The values for ethidium, 9-aminoacridine and amsacrine have been published previously (Wilson *et al.*, 1981*b*). All data are corrected for fluorescence quenching of the ethidium:DNA complex. For nitracrine this correction decreases the estimate of K by 8%.

[b]Input drug to DNA phosphate molar ratio at the equivalence point in the viscometric titration of PML-21 DNA.

[c]Bound drug to DNA phosphate molar ratio at the equivalence point in the viscometric titration of PML-21 DNA.

the neighbouring site exclusion model of McGee & Von Hippel (1974), but imply that the nitracrine site size may be atypically large in comparison with other aminoacridines. Site sizes of 3 or 4 base pairs would increase the estimated nitracrine association constant from $2.3 \times 10^5 M^{-1}$ to $3.8 \times 10^5$ and $7.2 \times 10^5 M^{-1}$ respectively. However, because the estimated proportion of bound drug decreases with increasing assumed site size, the estimated nitracrine unwinding angle increases by only 0.1 and 0.2° for site sizes of 3 and 4 base pairs respectively.

### Discussion

In choosing to evaluate the cytotoxicity of nitracrine under hypoxic and euoxic conditions we considered that the presence of a readily reduced nitro group on a heterocyclic nucleus might confer hypoxia-selective toxicity through a pathway similar to that for the nitroimidazoles (Brown, 1982). The predicted selective toxicity under hypoxia has been demonstrated (Figures 3 and 4). However, the properties of nitracrine in this experimental system differ in three respects from MISO.

1. Little or no threshold is evident prior to the onset of killing by nitracrine, in clear distinction to the kinetics of killing observed with MISO (Figure 2). If this shoulder with MISO is due to depletion of endogenous thiols (Varnes *et al.*, 1980; Taylor *et al.*, 1982) then such a process may be of lesser importance with nitracrine. Previous studies have indicated that reaction of nitracrine with DNA may be thiol-mediated (Gniazdowski *et al.*, 1975), so endogenous thiols could potentiate rather than protect from nitracrine toxicity. In addition, the low molar concentrations of nitracrine used, relative to MISO, makes it unlikely that direct titration of intracellular thiols will be of significance. Whatever the mechanistic basis for the observed difference between the two drugs, the rapid onset of killing by nitracrine may be an advantageous feature. In particular, if a large component of radiobiological hypoxia is due to a fluctuating blood supply (Brown, 1979) then exploitation of the resulting transient hypoxia will require agents with this characteristic.

2. After the initial shoulder, a constant first-order rate of killing by MISO is observed over the measurable range (Figure 1), while for nitracrine at low concentrations the rate of cell kill decreases markedly with time (Figure 4). The latter phenomenon could, in principle, be due to cell cycle stage selectivity or some other form of heterogeneity in responsiveness within the hypoxic cell population. However, the results illustrated in Figure 5 indicate a rapid loss of drug activity in hypoxic cultures, suggesting metabolic inactivation of nitracrine, or its sequestration by non-target binding sites. The difference between nitracrine and MISO need not reflect a different absolute rate of metabolism since the much higher concentration of MISO used would delay depletion. Loss of nitracrine is also observed under euoxic conditions (Figure 6), but as yet no direct comparison of the rate of metabolism under hypoxia and euoxia has been made.

3. Nitracrine is $\sim 10^5$-fold more potent than MISO as a hypoxic cell cytotoxic agent. Although the one-electron reduction potential ($E^1$) of nitracrine has not been reported, it is obvious that this compound will represent an anomaly with respect to the previously described correlation between $E^1 7$ and hypoxic cell toxicity for simple nitroheterocycles (Adams *et al.*, 1980).

The high potency of nitracrine is consistent with the hypothesis that the capacity of the acridine chromophore for intercalative binding to DNA might enhance the reactivity of reduced intermediates toward alkylation of DNA. It might be expected that the lack of planarity of the acridine chromophore in nitracrine, arising from steric interaction between the 1-$NO_2$ group and 9-dimethylaminopropylamino side chain (Dauter *et al.*, 1975), would decrease the propensity for intercalation. Furthermore, the density of binding sites in double-stranded DNA appears to be lower than that for other nitro- or aminoacridines (Filipski *et al.*, 1975), and nitracrine physically bound to DNA has less effect on template activity *in vitro* than have the isomeric derivatives with the nitro group in the 2, 3, or 4 position (Pawlak *et al.*, 1983). Despite this, the action of nitracrine in releasing and reversing supercoiling in closed circular DNA, shown by viscometric titration (Figure 7), provides convincing evidence for an intercalative binding mode. The unwinding angle of 16° is, in fact, similar ro that of the unsubstituted parent compound, 9-aminoacridine. These results confirm a previous report demonstrating intercalative binding on the basis of changes in sedimentation properties of circular DNA (Filipski *et al.*, 1977). The tolerance to substitution in the 1 and 9 positions with respect to intercalative binding suggests that metabolites of nitracrine, as well as the parent drug, will be able to bind to DNA by this mode.

Considerable evidence now suggests that the aerobic cytotoxicity of nitracrine is due to the formation of covalent DNA adducts (Konopa *et al.*, 1983; Pawlak *et al.*, 1983) rather than reversible physical complexes. The metabolism of nitracrine appears to be complex, with several competing routes available (Pawlak & Konopa, 1979). Reductive metabolism to the 1-hydroxylamine has been suggested to be responsible for the aerobic cytotoxicity of nitracrine (Konopa *et al.*, 1976), but the total covalent binding of labelled nitracrine to macromolecules in rat liver microsome preparations has been found to be *decreased* under hypoxia (Pawlak & Konopa, 1979). Metabolite(s) giving rise to protein adducts as observed in the latter study may differ from those responsible for cytotoxicity, which may act by inducing interstrand crosslinking of DNA (Konopa *et al.*, 1983). It should be noted that this apparent potential for bifunctional covalent reaction with DNA could provide an alternative explanation for the high dose potency of nitracrine relative to the nitroimidazoles, which appear to cause DNA strand scission (Palcic & Skarsgard, 1978; Taylor *et al.*, 1982) rather than interstrand crosslinking. The high potency of nitracrine under hypoxia suggests that evaluation of other chromophores offering both high DNA binding and high electron affinity is warranted. Such studies will help to clarify the hypothesized importance of physical DNA binding in the action of nitroheterocyclic drugs.

Preliminary results suggest that nitracrine is a potent radiosensitizer of hypoxic AA8 cells (W.R. Wilson, unpublished observations). The efficacy of this agent as a chemosensitizer of hypoxic cells also warrants investigation.

Despite clinical evidence of antitumour activity (Gniazdowski *et al.*, 1979) and an absence of myelosuppressive effects (Bratkowska-Seniow *et al.*, 1974), nitracrine has attracted little interest outside Poland. Whether the selective killing of noncycling hypoxic Chinese hamster cells in culture observed in the present study will also apply to hypoxic cells in human tumours remains to be determined. However, if nitracrine reaches such cells without excessive metabolic depletion, and if *in vivo* toxicity is not prohibitive, then its use in combination with treatment modalities which are limited by a sparing of hypoxic cells could provide a highly effective strategy for the treatment of solid tumours.

We wish to thank Mr R.J.B. Lambert and Ms S.M. Tapp for expert technical assistance, and Mrs K. Moustafa for typing the manuscript. This study was funded by the Cancer Society of New Zealand (National and Auckland Divisions).

### References

ADAMS, G.E., STRATFORD, I.J., WALLACE, R.G., WARDMAN, P. & WATTS, M.E. (1980). Toxicity of nitro compounds toward hypoxic mammalian cells *in vitro*: dependence on reduction potential. *J. Natl Cancer Inst.*, **64**, 555.

ALBERT, A. (1966). *The Acridines*. Edward Arnold and Son: London.

BAGULEY, B.C., DENNY, W.A., ATWELL, G.J. & CAIN, B.F. (1981). Potential antitumour agents. 34. Quantitative relationships between DNA binding and molecular structure for 9-anilino-acridines substituted in the anilino ring. *J. Med. Chem.*, **24**, 170.

BRATKOWSKA-SENIOW, B., OZYHAR-HAJPEL, H., RUNGE, I. & WAHL-MUGENSKA, M. (1974). Side effects of preparation C-283. *Arch. Immunol. Ther. Exp.* (*Warsz*), **22**, 411.

BROWN, J.M. (1979). Evidence for acutely hypoxic cells in mouse tumours, and a possible mechanism of reoxygenation. *Br. J. Radiol.*, **52**, 650.

BROWN, J.M. (1982). The mechanisms of cytotoxicity and chemosensitization by misonidazole and other nitroimidazoles. *Int. J. Radiat. Oncol. Biol. Phys.*, **8**, 675.

CAIN, B.F., ATWELL, G.J. & DENNY, W.A. (1975). Potential antitumour agents. 16. 4′-(acridin-9-ylamino)methane-sulfonanilides). *J. Med. Chem.*, **18**, 1110.

CAIN, B.F., BAGULEY, B.C. & DENNY, W.A. (1978). Potential antitumour agents. 28. Deoxyribonucleic acid polyintercalating agents. *J. Med. Chem.*, **21**, 658.

CHEN, T.R. (1977). *In situ* detection of mycoplasma contamination in cell cultures by fluorescent Hoechst 33258 stain. *Exp. Cell Res.*, **104**, 255.

CHODKOWSKI, J. & KIWAK, W. (1973). Polarographic investigations on acridine derivatives. Part III. Mononitro derivatives of 9-dimethylaminoalkylamino acridines. *Roczniki Chemii*, **47**, 157.

DAUTER, Z., BOGUCKA-LEDOCHOWSKA, M., HEMPEL, A., LEDOCHOWSKI, A. & KOSTURKIEWICZ, Z. (1975). Crystal and molecular structure of 1-nitro-9-(3-dimethylaminopropylamino-acridine (C-283) monoiodide. *Roczniki Chemii*, **49**, 859.

DENNY, W.A., BAGULEY, B.C., CAIN, B.F. & WARING, M.J. (1983). Antitumour acridines. In: *Molecular Aspects of Anticancer Drug Action* (Eds. Neidle & Waring). Macmillan: London, p. 1.

FILIPSKI, J., MARCZYNSKI, B. & CHORAZY, M. (1975). Complexes of derivatives of 1-nitro-9-aminoacridine with DNA. *Acta Biochim. Pol.*, **22**, 119.

FILIPSKI, J., MARCZYNSKI, B., SADZINSKI, L., CHALUPKA, G. & CHORAZY, M. (1977). Interactions of some nitro-derivatives of substituted 9-amino-acridine with DNA. *Biochim. Biophys. Acta*, **478**, 33.

GNIAZDOWSKI, M., SZMIGIERO, L., SLASKA, K., JAROSKAMINSKA, B. & CLESIELSKA, E. (1975). Influence of thiols on inhibition of ribonucleic acid synthesis *in vitro* by a 1-nitro-9-amino-acridine derivative, C-283. *Mol. Pharmacol.*, **11**, 310.

GNIAZDOWSKI, M., FILIPSKI, J. & CHORAZY, M. (1978). Nitracrine. In: *Antibiotics V/Part 2*, (Ed. Hahn). Springer-Verlag: Berlin. p. 275.

HALL, E.J. & ROIZIN-TOWLE, L. (1975). Hypoxic sensitizers: radiobiological studies at the cellular level. *Radiology*, **117**, 153.

HERSCHFIELD, V., BOYER, H.W., CHOW, L. & HELINSIKI, D.R. (1976). Characterization of a mini col $E_1$ plasmid. *J. Bacteriol.*, **126**, 447.

HIRST, D.G. & DENEKAMP, J. (1979). Tumour cell proliferation in relation to the vasculature. *Cell Tissue Kinet.*, **12**, 31.

KONOPA, J., CHOTKOWSKA, E., KOLDEJ, K., MATUSZKIEWICZ, A., PAWLAK, J.W. & WOYNAROWSKI, J.M. (1976). Studies on the mechanism of action of antitumour activity of ledakrin. *Mater. Med. Pol.*, **3**, 1.

KONOPA, J., PAWLAK, J.W. & PAWLAK, K. (1983). The mode of action of cytotoxic and antitumour 1-nitroacridines. III. *In vivo* interstrand cross-linking of DNA of mammalian or bacterial cells by 1-nitroacridines. *Chem.-Biol. Interact.*, **43**, 175.

KWASNIEWSKA-ROKICINSKA, C. & WINKLER, A.L. (1969). The effect of 1-nitro-9-aminoacridine on Yoshida sarcoma sensitive and resistant to alkylating agents. *Arch. Immunol. Ther. Exp.* (*Warsz*), **17**, 376.

LEDOCHOWSKI, A. & STEFANSKA, B. (1966). Research of tumour-inhibiting compounds. XXIX. Some $N^9$-derivatives of 1-, 2-, 3-, and 4-nitro-9-aminoacridine. *Ann. Soc. Chim. Polonorum*, **40**, 301.

LEVIN, V.A., WRIGHT, D.C., LANDAHL, H.D., PATLAK, C.S. & CSEJTEY, J. (1980). *In situ* drug delivery. *Br. J. Cancer*, **41**, (Suppl. IV), 74.

McGHEE, J.D. & VON HIPPEL, P.H. (1974). Theoretical aspects of DNA-protein interactions: co-operative binding of large ligands to a one-dimensional homogenous lattice. *J. Mol. Biol.*, **86**, 469.

MOORE, B.A., PALCIC, B. & SKARSGARD, L.D. (1976). Radiosensitizing and toxic effects of the 2-nitro-imidazole Ro-07-0582 in hypoxic mammalian cells. *Radiat. Res.*, **67**, 459.

OZOLS, R.F., LOCKER, G.Y., DOROSHOW, J.H., GROTZINGER, K.R., MYERS, C.E. & YOUNG, R.C. (1979). Pharmacokinetics of adriamycin and tissue penetration in murine ovarian cancer. *Cancer Res.*, **39**, 3209.

PALCIC, B. & SKARSGARD, L.D. (1978). Cytotoxicity of misonidazole and DNA damage in hypoxic mammalian cells. *Br. J. Cancer*, **37**, (Suppl. III), 54.

PAWLAK, J.W. & KONOPA, J. (1979). *In vitro* binding of metabolically activated ($^{14}$C)-ledakrin, or 1-nitro-9-$^{14}$C-(3′-dimethylamino-N-propylamino)acridine, a new antitumour and DNA cross-linking agent, to macromolecules of subcellular fractions isolated from rat liver and HeLa cells. *Biochem. Pharmacol.*, **28**, 3391.

PAWLAK, K., MATUSZKIEWICZ, A., PAWLAK, J.W. & KONOPA, J. (1983). The mode of action of cytotoxic and antitumour 1-nitroacridines. I. The 1-nitroacridines do not exert their cytotoxic effects by physicochemical binding with DNA. *Chem.-Biol. Interact.*, **43**, 131.

RADZIKOWSKI, C., LEDOCHOWSKI, Z., LEDOCHOWSKI, A., WRZOLEK, S., HRABOWSKA, M. & KONOPA, J. (1967). The evaluation of antitumour properties of acridine derivatives on the basis of the results from some *in vivo* and *in vitro* tests. *Arch. Immunol. Ther. Exp.* (*Warsz*), **15**, 126.

RADZIKOWSKI, C., LEDOCHOWSKI, A., HRABOWSKA, M. & 4 others. (1969). A search for antitumour compounds. VI. Biologic studies. Antitumour properties of 29 new acridine derivatives of groups XVII–XVIII. *Arch. Immunol. Ther. Exp.* (*Warsz*), **17**, 99.

RAUTH, A.M., PACIGA, J.E. & MOHINDRA, J.K. (1980). *In vivo* studies of the cytotoxicity of hypoxic cell radiosensitizers. In: *Radiation Sensitizers: Their Use in the Clinical Management of Cancer,* (Ed. Brady). Masson: New York. p. 207.

TANNOCK, I.F. (1968). The relation between cell proliferation and the vascular system in a transplanted mouse mammary tumour. *Br. J. Cancer,* **22,** 258.

TANNOCK, I. (1982). Response of aerobic and hypoxic cells in a solid tumour to adriamycin and cyclophosphamide and interaction of the drugs with radiation. *Cancer Res.,* **42,** 4921.

TANNOCK, I.F. & GUTTMAN, P. (1981). Response of Chinese hamster ovary cells to anticancer drugs under aerobic and hypoxic conditions. *Br. J. Cancer,* **43,** 245.

TAYLOR, Y.C. & RAUTH, A.M. (1980). Sulphydryls, ascorbate and oxygen as modifiers of the toxicity and metabolism of misonidazole *in vitro*. *Br. J. Cancer,* **41,** 892.

TAYLOR, Y.C., BUMP, E.A. & BROWN, J.M. (1982). Studies on the mechanism of chemosensitization by misonidazole *in vitro*. *Int. J. Radiat. Oncol. Biol. Phys.,* **8,** 705.

TEICHER, B.A., LAZO, J.S. & SARTORELLI, A.C. (1981). Classification of antineoplastic agents by their selective toxicities towards oxygenated and hypoxic tumour cells. *Cancer Res.,* **41,** 73.

THOMLINSON, R.H. & GRAY, L.H. (1955). The histological structure of some human lung cancers and the possible implications for radiotherapy. *Br. J. Cancer,* **9,** 539.

THOMPSON, L.H., FONG, S. & BROOKMAN, K. (1980). Validation of conditions for efficient detection of HPRT and APRT mutations in suspension-cultured Chinese hamster ovary cells. *Mutat. Res.,* **74,** 21.

VARGHESE, A.J. & WHITMORE, G.F. (1980). Binding of nitro-reduction products of misonidazole to nucleic acids and proteins. In: *Radiation Sensitizers,* (Ed. Brady). Masson: New York. p. 57.

VARNES, M.E., BIAGLOW, J.E., KOCH, C.J. & HALL, E.J. (1980). Depletion of non-protein thiols of hypoxic cells by misonidazole and metronidazole. In: *Radiation Sensitizers: Their Use in the Clinical Management of Cancer,* (Ed. Brady). Masson: New York. p. 121.

WANG, J.C. (1974). The degree of unwinding of the DNA helix by ethidium. I. Titration of twisted PM2 DNA molecules in alkaline caesium chloride density gradients. *J. Mol. Biol.,* **89,** 783.

WARING, M.J. (1976). DNA-binding characteristics of acridinyl-methanesulphonanilide drugs in comparison with antitumour properties. *Eur. J. Cancer,* **12,** 995.

WHILLANS, D.W. & RAUTH, A.M. (1980). An experimental and analytical study of oxygen depletion in stirred cell suspensions. *Radiat. Res.,* **34,** 97.

WILSON, W.R., GIESBRECHT, J.L., WHITMORE, G.F. & HILL, R.P. (1981*a*). Toxicity of 4′-(9-acridinylamino)methanesulfon-*m*-amside in exponential- and plateau-phase Chinese hamster cell cultures. *Cancer Res.,* **41,** 2809.

WILSON, W.R., BAGULEY, B.C., WAKELIN, L.P.G. & WARING, M.J. (1981*b*). Interaction of the antitumour drug 4′-(9-acridinyl-amino)methanesulfon-*m*-anisidide and related acridines with nucleic acids. *Mol. Pharmacol.,* **20,** 404.